# Supplementary material for: An allosteric transport mechanism for the AcrAB-TolC multidrug efflux pump
Source: eLife. 2017 Mar 29;6:e24905. doi: 10.7554/eLife.24905 (PMC5404916; doi:10.7554/eLife.24905)
Supplement: Supplementary file 1. — DOI: http://dx.doi.org/10.7554/eLife.24905.024 [file elife-24905-supp1.docx]

**Supplementary File 1**

**Table S1. CryoEM Data collection and processing**

|  | **AcrABZ-TolC with MBX3132** | **AcrABZ-TolC with Puromycin** | **apo AcrAB-TolC** |
| --- | --- | --- | --- |
| **Data collection** | | | |
| EM equipment | FEI Titan Krios | FEI Polara | FEI Titan Krios |
| Voltage (kV) | 300 | 300 | 300 |
| Detector | Gatan K2 | Gatan K2 | Gatan K2 |
| Super-resolution counting mode | yes | yes | no |
| Pixel size (Å) (after super-resolution) | 1.29 (0.65) | 1.62 (0.81) | 1.06 |
| Cumulative electron dose  (e/Å^2^) | 40 | 22 | 45 |
| Dose rate (electrons/pixel/sec) | ~8 | ~10 | ~5 |
| Exposure time (sec) | 8 | 6 | 10 |
| Defocus range  (μm) | 1.3~2.7 | 1.0~3.0 | 1.3~2.7 |
| Movie stacks (images) | 1,150 | 6,456 | 2,292 |
|  |  | | |
| **Data processing** |  | | |
| Defocus determining  software | CTFFIND4 | CTFFIND3 | GCTF |
| Motion correction software | MotionCorr | dosefgpu_driftcorr | MotionCor2 |
| Initial model software |  | EMAN2 |  |
| Refine software | RELION 1.4 | RELION 1.4 | RELION 2.0 |
| Particle picking software | RELION 1.4 | e2boxer.py | RELION 2.0 |
| Number of boxed Particles | 65,256 | 99,385 | 95,410 |
| Number of Particles used in reconstructions | 24,597 (TTT)  6,207 (TTL)  2,064 (TLL) | 20,349 (C1) | 13,544 |
| Symmetry | C3/C1 | C1 | C3 |
| Resolution (postprocess, Å) | 3.6 (TTT)  6 (LTT)  16 (LLT) | 5.9 (C1) | 6.5 |
| Map sharpening B-factor (Å^2^) | -70 | -150 | -213 |
