# Supplementary material for: An allosteric transport mechanism for the AcrAB-TolC multidrug efflux pump
Source: eLife. 2017 Mar 29;6:e24905. doi: 10.7554/eLife.24905 (PMC5404916; doi:10.7554/eLife.24905)
Supplement: Supplementary file 2 — DOI: http://dx.doi.org/10.7554/eLife.24905.025 [file elife-24905-supp2.docx]

**Supplementary File 2**

**Table S2   Model statistics of AcrABZ-TolC/MBX3132 pump**

| **Model composition of all proteins** | |
| --- | --- |
| Non-hydrogen atoms | 49,691 |
| Amino acid residues | 6,567 |
| **Refinement** | |
| Correlation coefficient | 0.76 |
| **Rms deviations** |  |
| Bonds (Å) | 0.0103 |
| Angles (^o^) | 1.31 |
| **Validation** | |
| Molprobity score | 2.06 |
| Clashscore, all atoms | 8.51 |
| Good rotamers (%) | 100 |
| **Ramachandran plot** | |
| Favored (%) | 87.95 |
| Allowed (%) | 11.97 |
| Outliers (%) | 0.08 |
