# Supplementary material for: An allosteric transport mechanism for the AcrAB-TolC multidrug efflux pump
Source: eLife. 2017 Mar 29;6:e24905. doi: 10.7554/eLife.24905 (PMC5404916; doi:10.7554/eLife.24905)
Supplement: Supplementary file 3. — DOI: http://dx.doi.org/10.7554/eLife.24905.026 [file elife-24905-supp3.docx]

**Supplementary File 3**

**Table S3. Crystallographic data and refinement statistics of AcrBZ complex with puromycin**

| Space group | P2_1_2_1_2_1_ |
| --- | --- |
| Cell dimensions |  |
| *a, b, c* (Å) | 147.25, 167.65, 249.98 |
| *α, β, γ* (^o^) | 90, 90, 90 |
| Resolution (Å) | 3.20 |
| R_merge_, % | 17.5 |
| Total number of observations | 262,652 |
| Unique reflections | 94,271 |
| Completeness, (outer shell)% | 92.5 (91.1) |
| I/σI (outer shell) | 5.2 (1.4) |
| <I> half-set correlation | 0.551 |
| B values Wilson plot/average in structure, Å^2^ | 36.2/66.7 |
| Reflections used for refinement | 89,522 |
| Geometry: Ramachandran favored, allowed, outliers, % | 97.15/2.51/0.34 |
| Rms deviation from ideal values for bond lengths (Å) and bond angles (^o^) | 0.0108/1.5487 |
| Number of non-hydrogen atoms in refinement |  |
| Refinement (R/R_free_ 5% reflections in test set), % | 19.54/24.89 |

The PDB ID for AcrBZ/puromycin structure is 5NC5
